# Salinity-Dependent Shift in the Localization of Three Peptide Transporters along the Intestine of the Mozambique Tilapia (*Oreochromis mossambicus*)

**DOI:** 10.3389/fphys.2017.00008

**Published:** 2017-01-23

**Authors:** Pazit Con, Tali Nitzan, Avner Cnaani

**Affiliations:** ^1^Agricultural Research Organization, Institute of Animal Science Rishon Letziyon, Israel; ^2^Department of Animal Sciences, Faculty of Agriculture, Food and Environment, The Hebrew University of Jerusalem Rehovot, Israel

**Keywords:** peptide absorption, PepT1, PepT2, slc15a1, slc15a2

## Abstract

The peptide transporter (PepT) systems are well-known for their importance to protein absorption in all vertebrate species. These symporters use H^+^ gradient at the apical membrane of the intestinal epithelial cells to mediate the absorption of small peptides. In fish, the intestine is a multifunctional organ, involved in osmoregulation, acid-base regulation, and nutrient absorption. Therefore, we expected environmental stimuli to affect peptide absorption. We examined the effect of three environmental factors; salinity, pH and feeding, on the expression, activity and localization of three PepT transporters (PepT1a, PepT1b, PepT2) along the intestine of the Mozambique tilapia (*Oreochromis mossambicus*). Quantitative real time PCR (qPCR) analysis demonstrated that the two PepT1 variants are typical to the proximal intestinal section while PepT2 is typical to the distal intestinal sections. Immunofluorescence analysis with custom-made antibodies supported the qPCR results, localized both transporters on the apical membrane of enterocytes and provided the first evidence for the participation of PepT2 in nutrient absorption. This first description of segment-specific expression and localization points to a complementary role of the different peptide transporters, corresponding to the changes in nutrient availability along the intestine. Both gene expression and absorption activity assays showed that an increase in water salinity shifted the localization of the PepT genes transcription and activity down along the intestinal tract. Additionally, an unexpected pH effect was found on the absorption of small peptides, with increased activity at higher pH levels. This work emphasizes the relationships between different functions of the fish intestine and how they are affected by environmental conditions.

## Introduction

The gills, intestine, and kidney are the main osmoregulatory organs in fish that respond to changes in environmental salinity. When fish move from freshwater to saltwater, the drinking rate accelerates in order to compensate for the spontaneous water loss to the hypertonic surrounding. Water and ions are absorbed at the intestine, the kidneys retain the water and the excess ions are actively secreted at the gills (Marshall and Grosell, 2006). The intestinal osmoregulatory function is tightly related to nutrient absorption, and both affect each other at the biochemical level (Ferreira and Baldisserotto, 2007). Ions act as the driving force for nutrient absorption from the intestinal lumen. Ion gradients are utilized to transport various nutrients into the intestinal epithelial cells (enterocytes) through pumps, transporters and channels (Thwaites et al., 2002; Bakke et al., 2010). The expression and activity of these absorption systems are under complex regulation in accordance with the various physiological functions of the intestine. It has been shown that expression of transporters changes in response to salinity alteration (Kalujnaia et al., 2007; Bucking and Schulte, 2012), and that the absorption of many important nutrients, such as glucose and different amino acids, is Na^+^-dependent (Ganapathy and Leibach, 1985). These relationships between nutrition and osmoregulation are more complex in euryhaline fish, experiencing frequent changes in environmental salinity (Wood and Bucking, 2011). With intestinal salinity adaptation of increased drinking, volume and rate of water inflow (Genz et al., 2008), adjustment of nutrient digestion and absorption is required. Therefore, salinity-dependent nutritional adaptations should be essential for euryhaline fish in order to thrive in a wide range of water salinities.

Protein is one of the most important nutrients in fish diets. Fish utilize proteins as a primary source for metabolism and energy needs, in addition to growth (Sire and Vernier, 1992). Protein utilization begins at the primary digestion site in the fish stomach (if not absent), continues in the intestine for final breakdown, and ends in absorption as free amino acids (FAA) and small peptides (di- and tri-peptides). The absorption of the final products of protein digestion is mediated by transport systems in the intestinal brush borders. FAA absorption is known to be mediated by different transporters, however, the only identified intestinal system for small peptide absorption is the Peptide Transporter (PepT) system (Verri et al., 2010).

PepT is a solute carrier and a member of the proton-dependent transporters family, coded by the SLC15A genes. It has been found in a wide range of organisms, from bacteria to humans (Leibach and Ganapathy, 1996; Daniel et al., 2006), including many fish species (Verri et al., 2003; Sangaletti et al., 2009; Terova et al., 2013; Hart et al., 2016). PepT is a co-transporter, moving small peptides into the enterocytes. This transporter has the capability to transport all potential 400 di-peptides and 8000 tri-peptides (Daniel and Kottra, 2004). This transport is coupled with proton entrance into the enterocytes. Thus, proton gradient at the apical membrane of the enterocyte is the driving force of the transport of small peptides (Verri et al., 1992). The necessity of the protons for the activity of PepT has been shown many times in different studies (Ganapathy and Leibach, 1983; Ganapathy et al., 1985; Thamotharan et al., 1996; Romano et al., 2014). In mammals, this driving force is considered to be maintained by the sodium-proton exchanger NHE3 (Ganapathy and Leibach, 1985; Thwaites et al., 2002). In teleosts, there have been contradicting reports regarding the participation of this exchanger in the maintenance of proton gradients (Kennedy et al., 2002; Verri et al., 2003). Another proton transport system found in the fish intestine is the vacuolar proton ATPase (V-H^+^-ATPase). This protein hydrolyzes ATP in order to excrete protons out of the cell and into the lumen (Guffey et al., 2011). Both transporters were thoroughly investigated in fish for their role in ion and pH regulation (Grosell et al., 2007, 2009; Grosell, 2011; Guffey et al., 2011), but not for their relationships with peptide transporters. We hypothesized that due to the PepT proton dependency, inhibition of these protons transporters will decrease proton availability, and result in lower PepT activity, as would be expected in high pH levels.

Two variants, PepT1 and PepT2, were found in mammals and other tetrapods, while in fish three PepT variants were found. The fish PepT1 has two variants, most likely a result of a second genome duplication specific to fish (Romano et al., 2014), PepT1a and PepT1b, coded by slc15a1a and slc15a1b (Gonçalves et al., 2007). This isoform has been characterized as a high capacity/low affinity transporter (Verri et al., 2003). The second isoform, PepT2, coded by the gene slc15a2 was identified as high affinity/low capacity transporter (Romano et al., 2006). Unlike mammals, in which PepT2 is expressed only in the kidney, expression of the fish PepT2 was shown at the transcript level in the gut as well (Romano et al., 2006), but the localization of the protein was never determined.

Mozambique tilapia (*Oreochromis mossambicus*), a tilapiine species of the family cichlidae, is a euryhaline teleost that thrives in a wide range of water salinities, from freshwater up to twice the seawater salinity. With these physiological capabilities, *O. mossambicus* emerges as a model for comparative research aimed at assessing salinity effects on fish physiology (Kültz et al., 2007). Bucking and Schulte (2012) described relationship between PepT1 and water salinity in three-spine stickleback (*Gasterosteus aculeatus*). In this work, our aim was to study environmental effects on PepTs in the *O. mossambicus* intestine. We hypothesized that changes in water salinity, that induce dramatic changes in expression levels of ion transporters due to the intestinal osmoregulatory adaptation (Seale et al., 2014; Ronkin et al., 2015), will affect the expression and activity of the intestinal peptide transporters, that depend on proton transporters. With the known differences in their transport kinematics (Romano et al., 2006), and transcriptome sequencing that showed expression of both PepT1 and PepT2 in the tilapia's intestine (Ronkin et al., 2015), we hypothesized that the transporters differ in their localization according to nutrients abundances, and will respond to environmental stimuli, to reflect a complementary role in small peptide absorption.

## Materials and methods

### Fish and handling

The experimental setup was described by Nitzan et al. (in press). Briefly, 36 Mozambique tilapia fish (97 ± 2.6 g, all males) were individually placed in aquaria within two closed recirculating systems (18 aquaria of 30 L in each system). Marine salt was gradually added to one of the systems to a final concentration of 3% (30 g/L). Fish were fed to satiety, once a day, at 8 a.m. All food residues were removed after 30 min. After 4 weeks, all fish were sacrificed and sampled at three time-points after feeding: 6, 24, and 72 h (six fish from each, freshwater and saltwater, each of the three time-points).

The intestine was removed from the abdominal cavity, cleaned from remaining connective tissues and fat, and food residues were gently removed. The intestine was divided into three sections: The anterior intestine (the region immediately following the stomach), middle intestine (the central intestinal section), and posterior intestine (the region preceding the anus). A one-cm long sample was collected from each section of each fish.

The study was approved by the ARO Committee for Ethics in Using Experimental Animals (Approval number: IL-210/09), and carried out in compliance with the current laws governing biological research in Israel.

### RNA extraction, cDNA synthesis

The dissected intestinal samples were immediately stored in RNAlater (Ambion), and kept at −20° until RNA extraction. Total RNA was extracted from the different tissues using Trizol reagent, purified from DNA contamination using TURBO DNA-free Kit (Ambion), quantified with Nano-Drop spectrophotometer (Thermo Scientific), and then reverse-transcribed into cDNA using the Verso cDNA Synthesis Kit (Thermo Scientific).

### Sequencing and phylogenetic analysis

In order to conduct phylogenetic analysis, the three *O. mossambicus* PepT cDNA variants were sequenced. Primer sets were designed for each gene (Appendix 1) based on the Genbank sequences of *O. niloticus* using the Primer 3 program (synthesized by HyLabs, Rehovot, Israel). The primers were used to amplify cDNA sequences from intestinal tissues, using a PCR reaction with a DreamTaq PCR Master Mix (Thermo Scientific). Following electrophoresis, the PCR amplicons were cleaned using PCRquick-spin kit (Intron Biotechnology), and sent for sequencing analysis (HyLab, Rehovot, Israel). Phylogenetic analyses were conducted using the MEGA (version 6) software (Tamura et al., 2013)

### Protein predicted structure

The predicted amino acid sequences were retrieved using the “Translate” tool of ExPASy: SIB bioinformatics resource portal. Trans-membranal domain prediction analysis was conducted on the predicted amino acid sequences using the TMHMM Server v. 2.0 (http://www.cbs.dtu.dk/services/TMHMM/). The TMRPres2D program (http://bioinformatics.biol.uoa.gr/TMRPres2D/) was used to construct a predicted secondary structure of the PepT proteins.

### Quantitative real-time PCR analysis

Forward and reverse primers for qPCR analysis were designed for each gene, using Primer 3 program (see Appendix 1). The primers were tested in all tissues using a PCR reaction. The primer sequences for NHE3 and V-H^+^-ATPase were taken from Hiroi et al. (2008). Elongation Factor 1 (EF-1) was used as a reference gene.

qPCR reactions were conducted using ABsolute™ Blue QPCR SYBR Green ROX Mix on an Eco Real-Time PCR System (Illumina). For each set of primers sequential 1:4 dilutions of cDNA mix were used to create standard curves, and the concentration for use as a template in the PCR reaction was chosen accordingly. Reaction efficiency was confirmed to be in the range of 82–99%. The data obtained from the qPCR were analyzed using the ΔCt method.

### Development of antibodies and western-blot analysis

Three different custom-designed antibodies were developed. The three protein sequences were analyzed for optimal antigenic regions. Three short peptides (PRKGSAESHKEDRRSSDSDDEC for PepT1a; C+SRQTLLIPSVISDEWLLTKDLTSKP for PepT1b; GYNVTHNKTVQRGEYTHATC for PepT2) were synthesized by Genemed Synthesis (San Antonio, TX, USA), and separately injected into rabbits. Antibodies were retrieved from the rabbits' blood after 3 months, purified by affinity purification and tested using ELISA by Genemed Synthesis. Western-blot analysis and immunofluorescence with immunizing peptides were conducted in order to test for specificity of recognition (Appendix 2).

### Immunofluorescence

Tissue samples from different intestinal sections were fixated in 4% paraformaldehyde (PFA) for 24 h followed by two washes in phosphate buffered saline (PBS) buffer and one wash of 50% ethanol (EtOH). The samples were then transferred to 70% EtOH and stored at 4°C.

Prior to creating paraffin blocks, the tissues were dehydrated through a series of graded EtOH baths to displace water (40 min of 70%, 96% and two washes with 100% of EtOH, followed by two Xylen baths for 40 min). Samples were embedded in paraffin and five micron sections were cut using a microtome and laid on microscope slides.

The slides were prepared for immunostaining by a series of washes with Xylen, decreasing EtOH concentration and PBS-T 0.05% buffer. Antigen retrieval was performed using citrate buffer (1.8 mM citric acid, 8.2 mM sodium citrate) heated to 100°C for 10 min. After three washes with PBS buffer, the slides were blocked for 1 h at room temperature with 300 μl of blocking solution (1% NGS, 1% BSA in PBS-T 0.05%). Following blocking, slides were incubated with the primary antibody solution, for 1 h at room temperature. Primary antibody solutions were prepared by dilution of the antibodies in blocking solution (1:200, 1:200, and 1:333 for rαPepT1a, rαPepT1b, and rαPepT2, respectively). The slides were washed three times in PBS-T 0.05% and incubated with the secondary antibody solution (goat α rabbit- Cy3 diluted 1:200 in blocking solution) for 1 h at room temperature in the dark. Following incubation, the slides were again washed with PBS-T 0.05% in the dark and stained with 150 μl of DAPI solution (Sigma) for nuclear staining (2.85 μM DAPI in PBS). Following a short rinse in PBS, the slides were covered with a cover glass and the stained sections were examined using a confocal microscope. In order to validate the specificities of the PepT antibodies, the antibodies were incubated with the immunizing peptides at 100-fold antibody concentration and the staining protocol was carried out in duplicates.

### Inverted gut sleeve technique

In order to examine the PepT transport activity in the intestine, a second experiment was conducted, in the same aquaria systems used for the first experiment. In this experiment, aquaria were stocked with 6 Mozambique tilapia fish (115.4 ± 9 g), three for each salinity. The rearing conditions, and salinization protocol of one of the systems, were similar to the first experiment. The fish were sampled 6 h after feeding.

Three intestinal sections (anterior, middle, and posterior) were dissected, and placed in ice-cold serosal solution (composition in mmol/L: NaCl 144, KCl 5.1, ${\text{CaCl}}_{2}^{*}$2H_2_O 1.4, ${\text{MgSO}}_{4}^{*}$7H_2_O 1.9, NaHCO_3_ 11.9, Na_2_HPO_4_ 2.9, glucose 5.5, pH 7) (Bucking and Schulte, 2012) until the beginning of the assay. Two gut sleeves were created of each section: Inverted and non-inverted, that served as control. The tissues were inverted by gently inserting a thin nylon thread into the intestine and tightening one edge of the intestine to the nylon thread with a silk thread. After the intestine was tightly anchored to the nylon thread, the intestine was inverted until all the lumen was exposed. Then, a small length of the inverted intestine was tied with silk thread, forming a closed sac. The non-inverted sac was tied in the same manner. The intestinal sacs were incubated in the appropriate radiolabeled mucosal solution, 0.5% solution (0.5% = 5 g of marine salt in 1L DDW) or 3% solution (3% = 30 g of marine salt in 1L DDW) for 20 min. Following the incubation, the tissues were quickly washed in clean serosal solution. The sizes of the sacs were calculated by measuring the length and width of the inverted sections. The tissues were transferred into 2 ml tubes with 0.5 mm glass beads (BioSpec) and homogenized using a mini-Beadbeater (BioSpec) in 600 μl of serosal solution. 4 ml of UltimaGold liquid scintillant (Perkin-Elmer,) were added to each homogenate, in 5 ml scintillation vials, before measuring scintillation in a beta-counter (Packard 1900 TR Scintillation Counter).

All solutions were aerated before the experiment. The final mucosal solutions were prepared by adding 100 μM non-radiolabeled di-peptide L-Ala-Ala (Sigma, A9502) to each (Bucking and Schulte, 2012). The 100 μM L-Ala-Ala stock was spiked with 10 μCi l^−1^ of [3H]-ala-ala (ART 0893-50 μCi). In order to convert scintillation to absorption rate, a standard curve was generated. 100 μM L-Ala-Ala serosal solution was spiked with 10 μCi l^−1^ of [3H]-ala-ala. A concentration curve was drawn from the scintillation reads, and used to interpolate the concentration of each sample.

### Inhibitors and pH effect

A third experiment was conducted in order to examine the effect of luminal pH on the activity of peptide transporters in the anterior intestine and to assess the peptide absorption dependency on two proton transporters; Vacuolar H^+^-ATPase (V-H^+^-ATPase) and the Na^+^/H^+^-Exchanger 3 (NHE3).

Three Mozambique tilapia fish were sacrificed by rapid decapitation 6 h after feeding. The intestine was removed from the abdominal cavity and connective tissue and fat were removed. The tissues were handled according to the inverted sleeve protocol described above. Seven sleeves were prepared from the anterior intestinal section, six inverted and one non-inverted for control. Each sleeve was incubated in different 0.5% mucosal solution; 100 μM L-Ala-Ala (sigma,) spiked with 10 μCi l^−1^ of [3H]-ala-ala, differing in their pH: 7, 8, and 9. In order to investigate the proton transporters' effect on the absorption, three mucosal solutions at pH 7 were prepared: 0.1 μM Bafilomycin A1 (V-H^+^-ATPas specific inhibitor) (InvivoGen), 50 μM EIPA- 5-(N-ethyl-N-isopropyl)-Amiloride (NHE3 specific inhibitor) (Cayman Chemical), and a third mucosal solution containing both inhibitors at the same concentrations. The inhibitor concentrations were chosen according to Brix and Grosell (2012) and Fenwick et al. (1999). Control, non-inverted sleeve was incubated in the pH 7 mucosal solution. Scintillation measurements and calculations of absorption rates were performed as described above.

### Statistical analysis

For the effects of time after feeding and salinity, statistical analyses were conducted separately for each of the three examined genes and for each intestinal section, using two-way analysis of variance (ANOVA), with time and salinity as variants, and fish as replicates. *Post-hoc* comparisons among groups were performed using the Tukey-Kramer HSD for the time and interactions effects, and student *t*-test for the salinity effect (α = 0.05). Correlations between expression levels of the examined genes were analyzed using Pearson correlation method, with individual fish as replicates (α = 0.05). Activity experiments were analyzed using one-way ANOVA. Data are presented as means ± SEM.

## Results

### Sequence analysis

**Figure 1:**
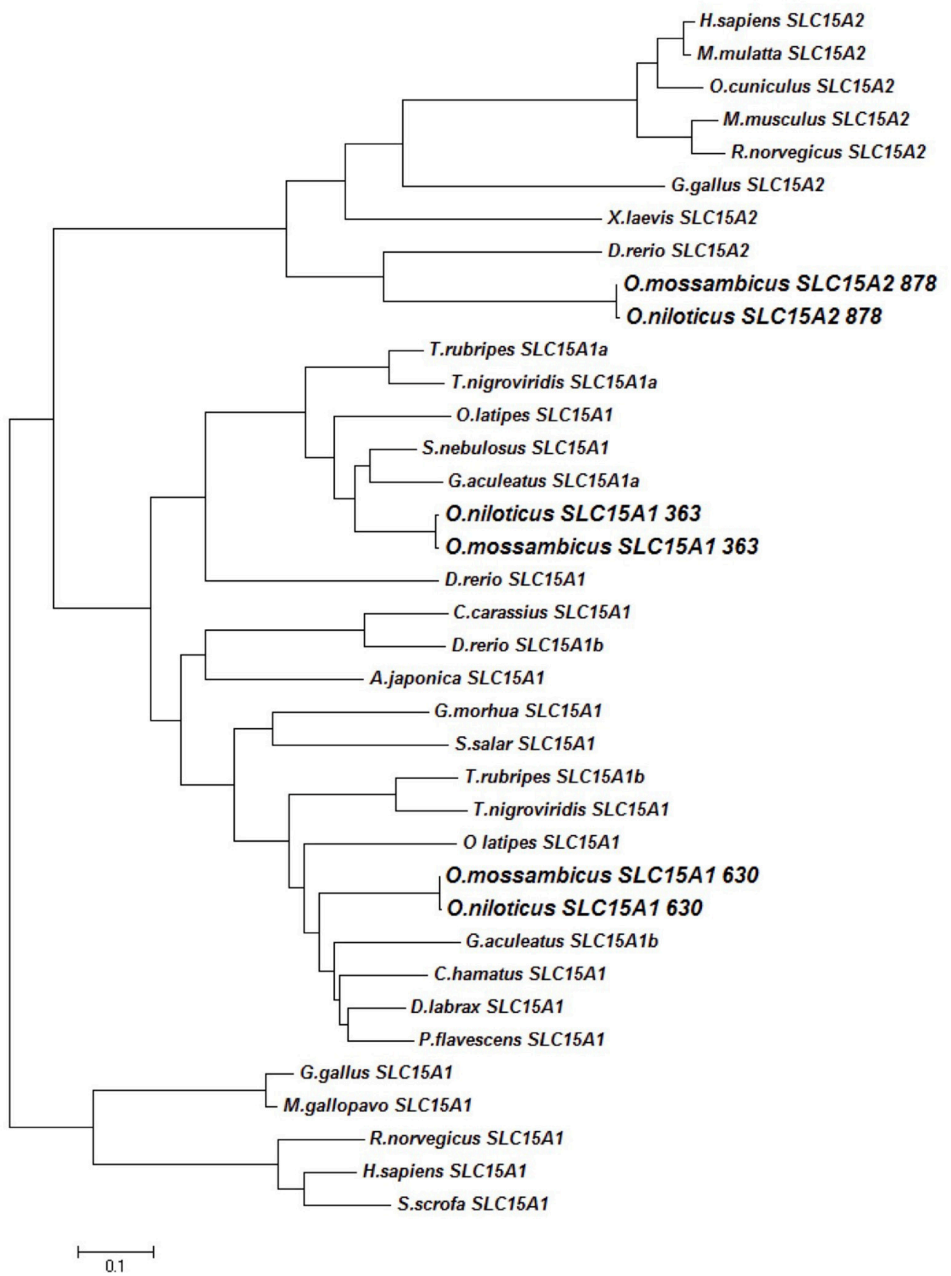
**Phylogenetic tree of SLC15A transcripts**. Genebank accessions numbers in Appendix 8.

Three slc15a variants were detected in the intestinal tract of the Mozambique tilapia, and were completely sequenced: slc15a1a (2958 bp), slc15a1b (3172 bp), and slc15a2 (4092 bp). The sequences were placed in the GenBank database under the accession numbers KX034110, KX034112, and KX034111 respectively. Phylogenetic analysis showed clustering of each variant with its Nile tilapia (*O. niloticus*) ortholog. Among vertebrates, two paralogous slc15a1 genes could only be found in fish, and both variants clustered with the mammalian and avian slc15a1 transcripts (Figure 1). The translated open reading frame (ORF) prediction was 725, 702, and 729 amino acids (AA) sequences for the slc15a1a, slc15a1b, and slc15a2 respectively. The Nile tilapia slc15a1b (LOC100691459) and slc15a2 (LOC100693193) are localized on LG16_21, while slc15a1a (LOC100706083) is on an unmapped scaffold. Shared synteny was found with the zebrafish genome, where the orthologs were mapped to chromosomes 9 and 6, respectively (Appendix 3).

The slc15a1 phylogenetic analysis showed a reverse clustering of the tilapia variants with the orthologs from other fish species, as the slc15a1a clustered with slc15a1b from other fish species, and vice versa (Figure 1). Nevertheless, the original name designation was kept for the rest of this paper. The tilapia slc15a2 transcript clustered with transcripts of other fish species within the phylogenetic branch of the vertebrate slc15a2.

### Structure analysis

**Figure 2:**
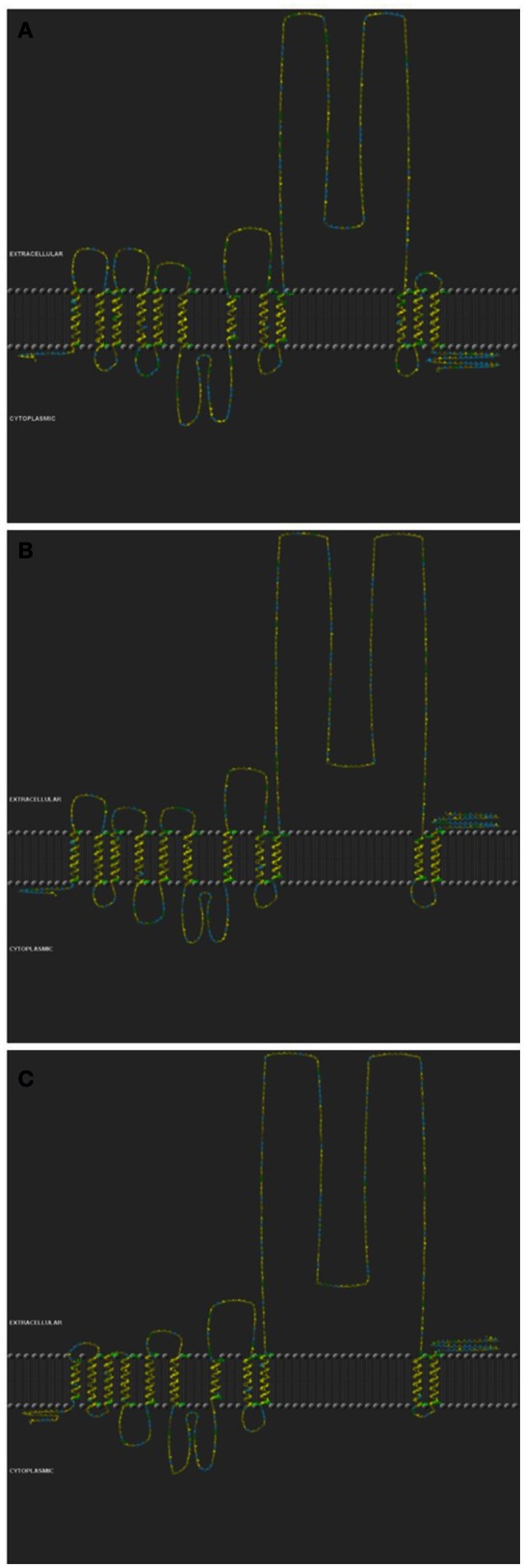
**Structure analysis of PepT1a (A)**, PepT1b **(B)**, and PepT2 **(C)** of the *Oreochromis mossambicus*. Color code indicate on Hydrophobic Potential', based on an *ad-hoc* hydrophobicity scale, by using a graduation from yellow (hydrophobic) to blue (hydrophilic).

The predicted secondary structure analysis showed a major difference between PepT1a to PepT1b and PepT2 (Figure 2). The PepT1a analysis predicted 12 trans-membranal helixes, whereas in the PepT1b and PepT2 there were only 11 predicted trans-membrane helixes. Due to this difference, the PepT1a C-terminus is predicted to be cytosolic, unlike the Pept1b and PepT2 C-terminals that are predicted to be extracellular.

### Gene expression

qPCR analysis showed section-dependent expression patterns for the three intestinal PepT genes. slc15a1a and slc15a1b were expressed only in the anterior and the middle intestine, whereas slc15a2 expression was found in the middle and posterior sections.

**Figure 3:**
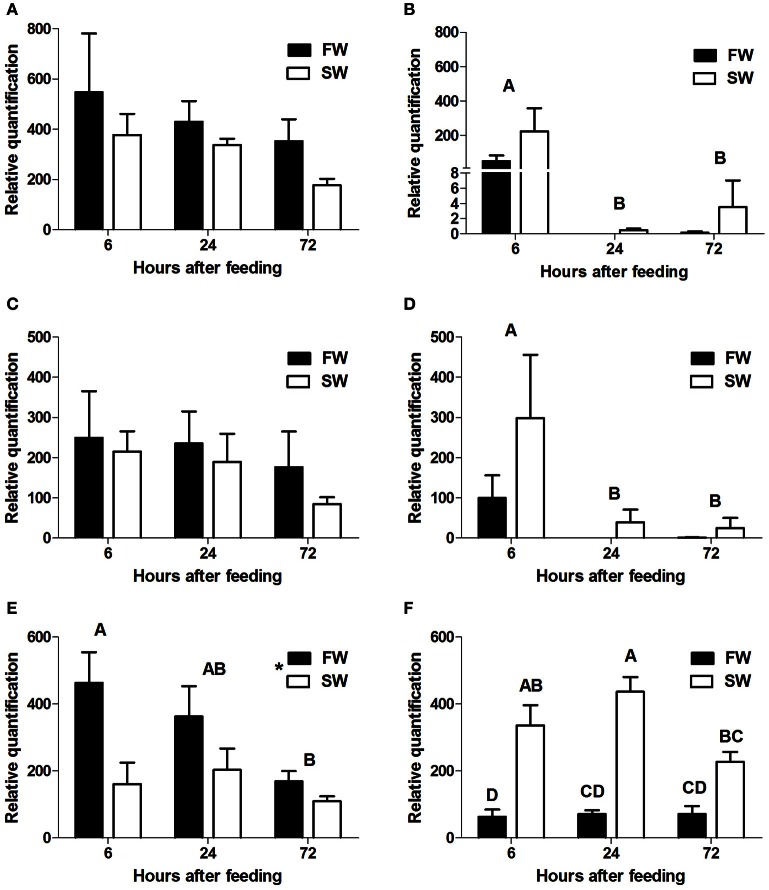
**Relative quantification of mRNA expression of slca15a1a in the anterior (A)** and middle **(B)** intestine, slc15a1b in the anterior **(C)** and middle **(D)** intestine, and slc15a2 in the middle **(E)**, and posterior **(F)** intestine. AI, MI, and PI stand for anterior, middle, and posterior intestine, respectively. Different letters indicate significant differences between time-points after feeding, except **(F)** where there was significant interaction between time and salinity. In **(E)** the asterisks indicate on significant difference between salinities (*N* = 6 for each treatment).

In the anterior intestine, both slc15a1 variants showed a non-significant trend of decreasing levels of expression, over the time course (Figures 3A,C). In the middle intestine, there was a significant decrease in both variants at the 24 h post-feeding time point (*P* = 0.004, *P* = 0.01). There were no significant differences between the salinity treatments for either of the variants (Figures 3B,D).

Transcript levels of slc15a2 in the middle intestine showed a significant decrease (*P* = 0.04) from 6 to 72 h post-feeding, and transcript levels were significantly higher (*P* = 0.004) in FW than in SW (Figure 3E). Significant interactions (*P* = 0.024) between time and salinity were observed in the posterior intestine. In SW there was an increasing trend between 6 and 24 h, followed by a decrease at the 72 h time-point. In FW, there was no significant difference between the three time-points. Unlike the middle intestinal sections, transcript levels in the posterior intestine were significantly higher in SW than in FW at 6 and 24 h post feeding (Figure 3F). Relative expression levels of VHA and NHE3 were measured (Appendix 4), and comparison of the expression patterns showed significant correlations between the PepT genes and the V-H^+^-ATPase (Table 1).

**Table 1:** **Significant correlations between expressed genes along the intestinal segments, in two water salinities**.

**Tissue**	**Anterior interior**			**Middle intestine**			**Posterior intestine**		
	**Correlation**	***r***	***p*****-value**	**Correlation**	***r***	***p*****-value**	**Correlation**	***r***	***P*****-value**
FW	PepT1a X PepT1b	0.75	0.0004	PepT1a X PepT1b	0.89	<0.0001			
	VHA X PepT1a	0.87	<0.0001	VHA X PepT2	0.58	0.0109			
	VHA X PepT1b	0.65	0.0049						
	VHA X NHE3	0.54	0.026						
SW				PepT1a X PepT1b	0.93	<0.0001	VHA X PepT2	0.64	0.0057
				VHA X PepT1a	0.52	0.0452			
				VHA X PepT1b	0.56	0.0225			

*VHA stands for V-H^+^-ATPase*.

### Protein expression

**Figure 4:**
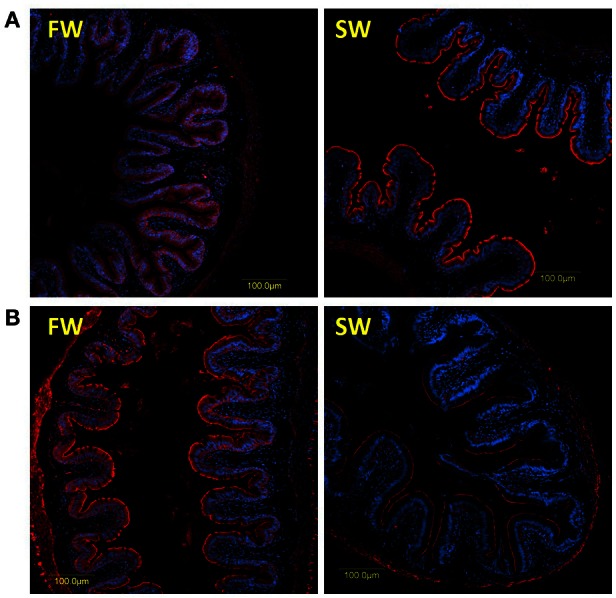
**Immunofluorescence staining (anti PepT in red and DAPI in blue) of the middle intestine with rabbit anti PepT1b (A)** showing higher expression in SW, and rabbit anti PepT2 **(B)** showing higher expression in FW.

**Figure 5:**
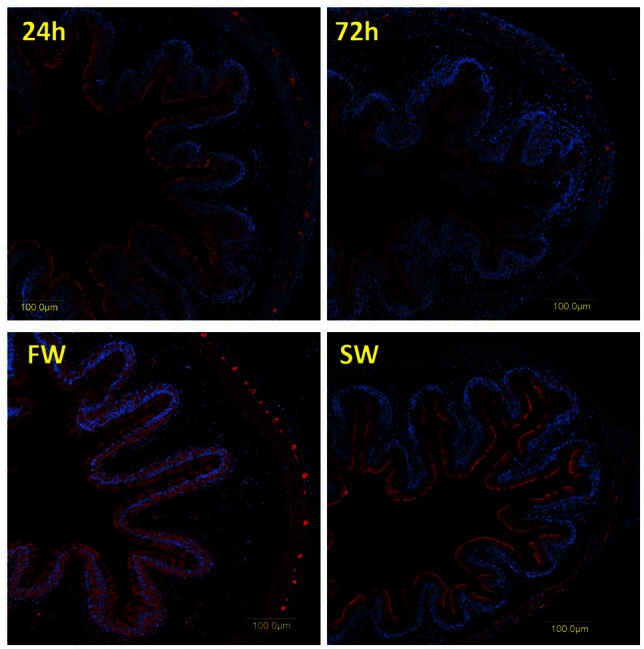
**Immunofluorescence staining of the posterior intestine with rabbit anti PepT2 (red) and DAPI for nuclei (blue)**.

The specificities of the antibodies produced for the three tilapia PepT isoforms were demonstrated in Western blot analysis (Appendix 2). Both PepT1 and PepT2 antibodies showed complete elimination of the signal when incubated with the immunizing peptide in an immunofluorescence assay (Appendix 5). This result indicates specific staining and rejects non-specific recognition of the different variants. Immunofluorescence assays showed strong signals at the apical membrane for all three antibodies, and none at the basolateral side of the enterocytes (Figures 4, 5). In the anterior intestinal section, staining was only observed with PepT1a and PepT1b antibodies, whereas in the middle intestinal section, staining was observed with all three PepT antibodies. In the posterior intestinal section, the only detected staining was with the PepT2 antibody. Thus, the protein localization of the PepT variants was in accordance with the results of the gene expression analysis.

A clear difference between the 6 and 72 h post-feeding time-points was observed in the anterior and middle intestinal sections, showing a decreasing signal for both PepT1a and PepT1b over time. A salinity effect was observed in all three intestinal sections (Figures 4, 5 and Appendix 6). At the posterior intestine, there was a clear decrease in signal over time in saltwater (Figure 5).

### Absorption activity

**Figure 6:**
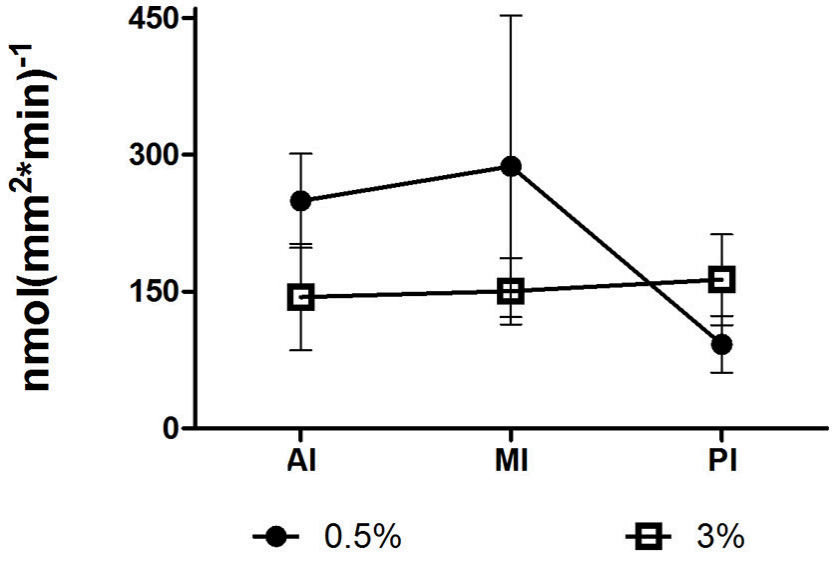
**Absorption rate of L-Ala-Ala along the intestine, in saltwater, and brackish water, at 6 h after feeding time point**. AI, MI, and PI stand for anterior, middle, and posterior intestine, respectively.

An activity assay was conducted using the inverted gut-sleeve technique. The absorption of L-Ala-Ala was measured using a radiolabeled di-peptide solution in which the tissues were incubated. At 6 h after feeding, the absorption rate was lower at SW in the anterior and middle intestinal sections and higher in the posterior intestinal section (Figure 6). Although these differences did not reach the *p* < 0.05 statistical significance threshold, the salinity effect trends seen for absorption rate were of a similar pattern to the salinity effect found for the PepT gene expression, measured by qPCR in the anterior and posterior intestinal sections (Figures 3, 6).

**Figure 7:**
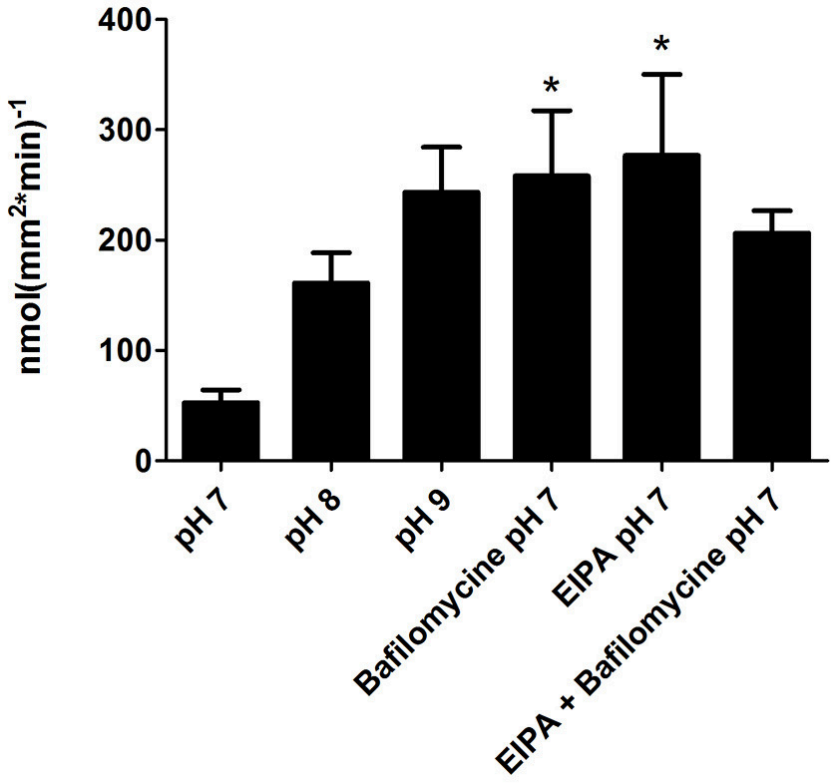
**Absorption rate of L-Ala-Ala at different pH and in the presence of two proton transporters inhibitors; Bafilomycine (V-H^**+**^-ATPasspecific inhibitor), and EIPA (NHE3 specific inhibitor), in the anterior intestinal section**. Asterisks indicate on a significant difference in compare to the pH 7 treatment.

The pH effect on di-peptide absorption was examined using the same activity assay, conducted at different pH levels, in the presence of inhibitors of two proton-transporters (V-H^+^-ATPase and NHE3), at the anterior intestinal section. Treatments with each one of the inhibitors resulted in a significantly higher absorption rate, compared to the pH 7 treatment (*P* = 0.021; *P* = 0.034). An increased absorption rate of L-Ala-Ala was seen along with the elevated pH of the mucosal solutions (with *P* = 0.0503 for pH 9). When added together to the mucosal solution, the inhibitors had a lower effect, compared to their individual effects (Figure 7).

## Discussion

PepTs have been suggested to play an important role in the fish protein supply (Bogé et al., 1981; Reshkin and Ahearn, 1991; Dabrowski et al., 2003). We found three PepT coding genes in the Mozambique tilapia that are expressed in the intestine and show a high degree of similarity to their homologs in the Nile tilapia, indicating high conservation between these two species.

The difference in the number of trans-membrane domains (TMD), found in the secondary structure prediction, along with the vertebrate phylogenetic analysis are an indication of the role of genome duplications in the evolution of these genes. The two variants of PepT1, that probably resulted from the teleost genome duplication (Gonçalves et al., 2007; Romano et al., 2014; and Appendix 3), differ in the number of their TMD. The extra TMD and the cytosolic carboxyl terminal of PepT1a are possibly an adaptation leading to intracellular signaling pathway. To the best of our knowledge, this variation was not previously characterized or analyzed. A comparative analysis showed an identical pattern in *G. aculeatus* and *Tetraodon nigrovirdis* (Appendix 7). The structural variation between PepT1 genes suggest that other than luminal nutrient sensing, the fish PepT system activity is governed by interactions with cytosolic factors.

The immunofluorescence analyses demonstrated that PepTs are localized at the intestinal brush borders. This exclusive localization on the apical membrane of the enterocytes indicate, that if it exists, transport of di- and tri-peptides at the basolateral membrane is mediated by other transporters, as shown in the mammalian Caco-2 cell monolayers (Saito and Inui, 1993; Terada et al., 1999). Alternatively, the di-peptides can be cleaved in the cell into FAAs, that are then transported through the basolateral membrane.

This is the first work showing immune-localization of PepT2 at the gastrointestinal tract of fish. Romano et al. (2006) have shown before that this transporter transcript is expressed in the zebrafish intestine. However, the authors suggested that the localization of the transporter may be similar to the mammalian PepT2, which associates to neuromuscular layers in the gastrointestinal tract (Rühl et al., 2005). Here, we have shown a specific immunofluorescence signal for PepT2 at the intestinal brush borders of the Mozambique tilapia.

The segment-specific expression, seen at both the mRNA and protein levels, points to a complementary role of the different peptide transporters, corresponding to the changes in nutrient availability along the intestine. The PepT1 variants, which are high capacity/low affinity transporters, are expressed at the proximal sections where there are high levels of peptides obtained from the feed. The PepT2 variant, which is a low capacity/high affinity transporter (Romano et al., 2006), is highly expressed at the distal section of the intestine, where it can retain the absorption rate of small peptides at lower substrate concentrations. While PepT1 had been widely studied in fish in relation to protein utilization (Dabrowski et al., 2003; Hakim et al., 2009; Terova et al., 2013), there is little work on PepT2. In mammals PepT2 is mainly associated with the re-absorbance of peptide in the kidney (Silbernagl et al., 1987; Daniel and Kottra, 2004). Our findings show specific adaptations of intestinal sections that can maximize protein utilization and absorption from the lumen.

Section-specific expression was also found when analyzing the time after feeding effect. The three time points in which the fish were sampled allowed tracking of the complete digestive process. Transcript expression changed in correlation with the availability of the food along the intestine; highest at 6 h after feeding at the anterior and middle intestine, and at 24 h after feeding at the posterior intestine.

**Figure 8:**
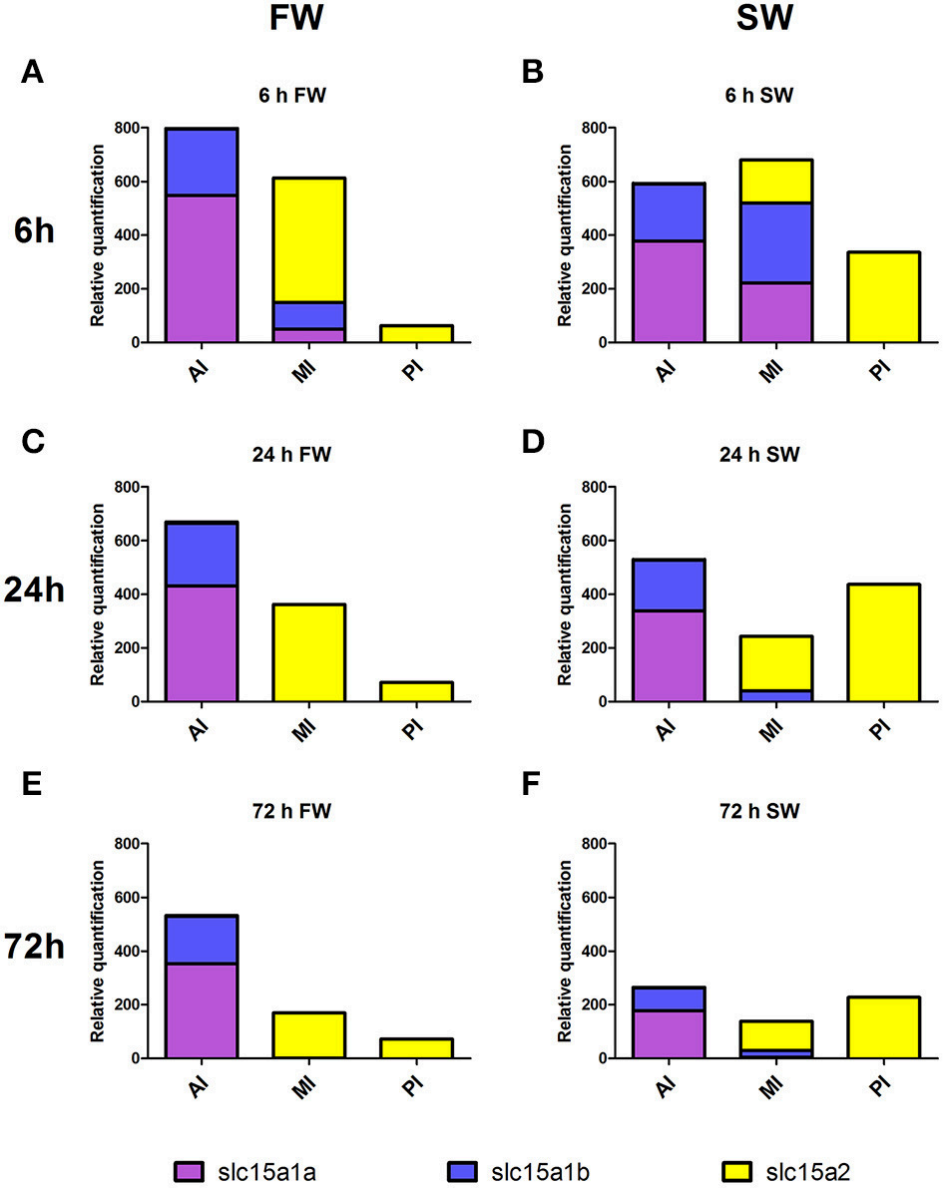
**Expression of the three PepT variants along the intestine at FW (A,C,E)** and SW **(B,D,F)**, at different time-points after feeding. AI, MI, and PI stand for anterior, middle, and posterior intestine, respectively.

In order to gain a broad view on the expression of peptide transporters the relative quantification of all three PepT genes were plotted together (Figure 8). This graph is a visual presentation of the expression changes along the intestinal segments, for each of the three PepT genes, demonstrating a salinity-dependent localization shift toward the distal sections of the intestinal tract. For example, this shift can be seen at 6 h after feeding, with PepT1a and PepT1b genes having about 5-fold higher expression in the anterior intestine than in the middle intestine in freshwater, and almost equal expression in saltwater. Similarly, the PepT2 gene showed higher expression in the middle intestine than in the posterior intestine in freshwater, while the reverse was seen in saltwater (Figures 8A,B). Resulting from this shift, a uniform dispersion of transporters expression along the intestine was seen in saltwater compared to the steady decline seen in freshwater.

The salinity-dependent shift was also seen when analyzing the correlation between the expression of the PepT genes and V-H^+^-ATPase. These data imply that V-H^+^-ATPase might have a role in the supply of protons for the tilapia intestinal absorption of small peptide through the PepT systems, as an alternative or complement to NHE3 activity known in mammals. Until now, many lines of evidence have been presented for the co-expression and activity of NHE3 with small peptide absorption (Ganapathy and Leibach, 1985; Kennedy et al., 2002; Thwaites et al., 2002; Daniel, 2004), while the V-H^+^-ATPase was mainly known for its role in intestinal pH regulation and Cl^−^ absorption (Guffey et al., 2011). To the best of our knowledge, this is the first study to show a connection between V-H^+^-ATPase and the PepT systems. Given that small peptide absorption is pH-dependent and requires an inwards proton gradient, it is reasonable to assume a role for the V-H^+^-ATPase in the proton gradient maintenance.

The absorption rates of L-Ala-Ala along the intestine showed a pattern similar to the gene expression; as well as a salinity-dependent shift of absorption activity down the intestinal tract. In the anterior and posterior intestine, the effects of salinity on gene expression and absorption activity were very similar. While this was not the case in the middle intestine, it should be noted that in the middle intestine there was a notable change in the composition of PepT genes that were expressed, shifting from dominantly PepT2 in freshwater to PepT1 in saltwater. Unlike the qPCR analysis, where the three different genes could be distinguished from one another, the activity assay measures the combined absorption of all PepT systems. It is known that in zebrafish, PepT2 exhibits high-affinity/low-capacity transport activity, while PepT1 exhibits low-affinity/high-capacity transport activity (Verri et al., 2003; Romano et al., 2006). Therefore, the higher absorption in freshwater may be due to the higher expression of the high-affinity PepT2. Further investigation is required to better understand the kinematic properties of the three PepT variants and enable an accurate correlation between gene expression and protein activity.

The phenomena of salinity-dependent localization shift toward the distal sections of the intestinal tract could be due to the fish higher drinking rate in saltwater than in freshwater, resulting in rapid movement of nutrients down the intestine (Bucking et al., 2011; Grosell, 2011; Lin et al., 2013). Alternatively, this could be a result of a complex regulation of the interacting osmoregulatory and nutritional functions of the intestine. Thus, changes in luminal ion composition, pH, and the substrate concentrations, will affect the PepT expression and activity (Verri et al., 2010). Whether this shift is due to the physical differences in nutrients availability in the lumen, or results of intrinsic regulation in response to salinity change, the ability of euryhaline fish to modify the various interacting intestinal functions should be of importance to its fitness under a wide range of water salinities.

We examined the effect of direct pH changes and inhibitors of NHE3 and V-H^+^-ATPase on PepT activity. We expected that with the increase in pH, there will be a subsequent decrease in the absorption rate, due to the total or partial elimination of the proton gradient. However, we found a dose response trend of an increased absorption rate with increased pH of the mucosal solution. A similar effect was seen when the inhibitors were added to the mucosal solution at pH 7. Considering the proton gradient dependency of the PepT activity, these results were not expected. A similar phenomenon was detected in zebrafish by Verri et al. (2003) using the two-electrode voltage clamp technique. This phenomenon was never reported in mammalian peptide transporters, that showed optimal absorption at pH 6, with a decrease at a more basic environment (Ganapathy and Leibach, 1983). Verri et al. (2003) suggested that this is a unique characteristic of the zebrafish transporter, adapted to an alkaline surrounding, due to the absence of a stomach in zebrafish. However, contrary to the zebrafish, the Mozambique tilapia does have a stomach, thus there should be some other explanation for these results. In their work, Verri et al. (2003) showed that in an alkaline surrounding, the transporter capacity increased while the affinity of the transporter decreased. Therefore, it is possible that the pH-dependent increase in di-peptide transport that we observed was due to an increase in the transporter capacity at the specific substrate concentration that we used. This mechanism can also explain the apparent contradiction between the results of the activity assay with the proton transporters inhibitors and the correlations found between the expression of PepT and V-H^+^-ATPase (Table 1). Along with being the driving force for peptide transport, protons contribute to the affinity of the transporter (Verri et al., 2003). Hence, the V-H^+^-ATPase could be necessary to maximize small peptide absorption in varying substrate concentrations along the intestine. Further investigation is required to understand the complex relationships between the intestinal peptide and proton transporters.

In this work, we characterized the genetic sequence, the secondary structure, and the tissue localization of the tilapia's PepT systems. We showed for the first time that PepT2 is expressed at the brush borders of the Mozambique tilapia, and demonstrated a specific expression of PepT isoforms between the intestinal sections corresponding to lumen substrate concentrations. Salinity and time after feeding affected these transporters and their supporting systems, mainly on their localization along the intestine. An unexpected effect of pH on small peptide absorption was found, with increased activity at higher pH levels. The data presented in this study are a further indication of the strong connection between feeding and osmoregulation, and can be important for research on protein absorption and utilization.

## Author contributions

PC and AC conceived and designed the experiments. PC, TN, and AC challenged the animals and sampled tissues. PC and TN performed the RNA analyses. PC performed the computational analyses, immunofluorescence and activity assays. PC and AC wrote the manuscript.

## Funding

This research was supported by Research Grant No. IS-4296-10 from BARD, The US-Israel Binational Agricultural Research and Development Fund, and by grants 356-0672 and 356-5469 from the Chief Scientist of the Ministry of Agriculture and Rural Development.

### Conflict of interest statement

The authors declare that the research was conducted in the absence of any commercial or financial relationships that could be construed as a potential conflict of interest.
