# Corrigendum: Salinity-Dependent Shift in the Localization of Three Peptide Transporters along the Intestine of the Mozambique Tilapia (*Oreochromis mossambicus*)

**DOI:** 10.3389/fphys.2020.00062

**Published:** 2020-02-06

**Authors:** Pazit Con, Tali Nitzan, Avner Cnaani

**Affiliations:** ^1^Agricultural Research Organization, Institute of Animal Science, Rishon Letziyon, Israel; ^2^Department of Animal Sciences, Faculty of Agriculture, Food and Environment, The Hebrew University of Jerusalem, Rehovot, Israel

**Keywords:** peptide absorption, PepT1, PepT2, slc15a1, slc15a2

In the original article, in Appendix 1 there was a mistake in “Primers sets for qPCR” as published. The names (only) of the Primers sets of the SLC15A1b and SLC15A2 were mixed and appear incorrectly in the table.

The corrected “Primers sets for qPCR” appears below.

The authors apologize for this error and state that this does not change the scientific conclusions of the article in any way. The original article has been updated.

**Table:** Primers sets for qPCR.

**Gene**	**GeneBank**	**Forward**	**Reverse**
SLC15A1a	XM_003459630	TAAAACCCTGCCTGACTTCC	AATCCTCATTAGCCCCAAAA
SLC15A1b	XM_003447363	CCAAGCCAGAACAAGGTAACA	GGCTCAATTAGTCCCAAGTCC
SLC15A2	XM_003454878	CTGCGAACGCTTCTCCTACT	CGCTGAAAGCATGGTAGACA
NHE3	AB326212.1	AAGCGGCACCCATCACTACA	GAGCCAGCAAACCAGAATCCA
H^+^-ATPase	AB369668	CCACAGCTCAGAGCGACAACA	GCATACTCGGCCTTGATCTTG
EF1	XM_003458541	TCAACGCTCAGGTCATCATC	ACGGTCGATCTTCTCAACCA

