# Supplementary material for: Salinity-Dependent Shift in the Localization of Three Peptide Transporters along the Intestine of the Mozambique Tilapia (Oreochromis mossambicus)
Source: Front Physiol. 2017 Jan 23;8:8. doi: 10.3389/fphys.2017.00008 (PMC5253378; doi:10.3389/fphys.2017.00008)
Supplement: Supplementary file 1 [file DataSheet1.DOCX]

# Appendix 1

|  | **set** | **Forward** | **Reverse** |
| --- | --- | --- | --- |
| **SLC15A1a**  **XM_003459630** | **1** | **GCCATGGCAGTTGAAAAGAT** | **AACCCTGCTGGTCAAAGAGA** |
|  | **2** | **CACGTCCAGCAAGAGTGCTA** | **GCAAGGAAGCGAAGAGAATG** |
|  | **3** | **CTGTGCGTGACAAGGTGTTC** | **CAGAGCATTGGAGCATTGAA** |
| **SLC15A1b**  **XM_003447363** | **1** | **ACAGGAAACAGTCCCACTCC** | **AAAGCCATAGCAGCCAGAAA** |
|  | **2** | **GCTGCAAGCTACCACAATGA** | **GCTGCAAGCTACCACAATGA** |
|  | **3** | **CGTCCTTTTTGCGTCTCTCT** | **CGTCCTTTTTGCGTCTCTCT** |
| **SLC15A2**  **XM_003454878** | **1** | **GCTGCTTTTCCTGAGTGAGC** | **GCAGCGTCAGGGTAAATGAT** |
|  | **2** | **TGAACCTGGCTTTTGGAAAC** | **TGGAGAAGATGATGCAGACG** |
|  | **3** | **CATCACTGGCCTGGAGTTCT** | **TCCTCCTGTTTTCCCACGTA** |
|  | **4** | **GCATGTAGTGCAGCCAGTGT** | **TGAAGTGTCCCAATGAGCAG** |

Primers sets for sequencing analyses.

Primers sets for qPCR.

| **Gene** | **GeneBank** | **Forward** | **Reverse** |
| --- | --- | --- | --- |
| **SLC15A1a** | **XM_003459630** | **TAAAACCCTGCCTGACTTCC** | **AATCCTCATTAGCCCCAAAA** |
| **SLC15A1b** | **XM_003447363** | **CCAAGCCAGAACAAGGTAACA** | **GGCTCAATTAGTCCCAAGTCC** |
| **SLC15A2** | **XM_003454878** | **CTGCGAACGCTTCTCCTACT** | **CGCTGAAAGCATGGTAGACA** |
| **NHE3** | **AB326212.1** | **AAGCGGCACCCATCACTACA** | **GAGCCAGCAAACCAGAATCCA** |
| **H^+^-ATPase** | **AB369668** | **CCACAGCTCAGAGCGACAACA** | **GCATACTCGGCCTTGATCTTG** |
| **EF1** | **XM_003458541** | **TCAACGCTCAGGTCATCATC** | **ACGGTCGATCTTCTCAACCA** |
