# Supplementary material for: Salinity-Dependent Shift in the Localization of Three Peptide Transporters along the Intestine of the Mozambique Tilapia (Oreochromis mossambicus)
Source: Front Physiol. 2017 Jan 23;8:8. doi: 10.3389/fphys.2017.00008 (PMC5253378; doi:10.3389/fphys.2017.00008)
Supplement: Supplementary file 2 [file DataSheet2.DOCX]

**Appendix 2**

Total proteins extraction and Western- blot protocols:

Total proteins were extracted using SEID buffer with 1:100 protease inhibitor cocktail (Sigma) and the concentration was determined using Bradford assay. Samples were prepared for SDS-PAGE separation and transfer to a nitrocellulose. Afterwards, membranes were stained with Ponceau S solution for total protein staining to confirm equal loading and transfer. The membranes were blocked with 5% skim milk (PBS-T 0.05%) following with incubation with the primary antibody, Rabbit anti PepT (rαPepT;) in 1% BSA PBS solution (1:150, 1:150, 1:300 for rα PepT1a, rα PepT1b and rα PepT2 respectively) for 1.5 hours at room temperature. The membrane were washed three times in 5% skim milk (PBS-T 0.05%) to remove excess of antibodies, transferred to 1 hour incubation in secondary antibody solution (1:3,000 goat anti rabbit HRP conjugated antibody in 5% skim milk) followed with three washes. For chemiluminescence development, a SuperSignal™ West Dura Extended Duration Substrate Kit (Thermo scientific,) was used. The membranes were incubated in the solution for 2 minutes and an image was capture using GBox gel imager (Sygene) (appendix 7).

Western blot assay for specific interactions of three anti- PepT variants antibodies: PepT1a (A), PepT1b (B) and PepT2 (C).


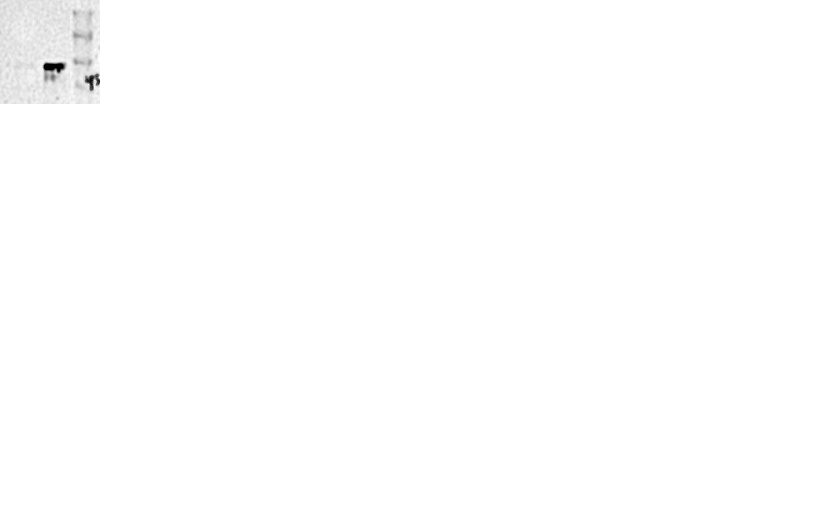

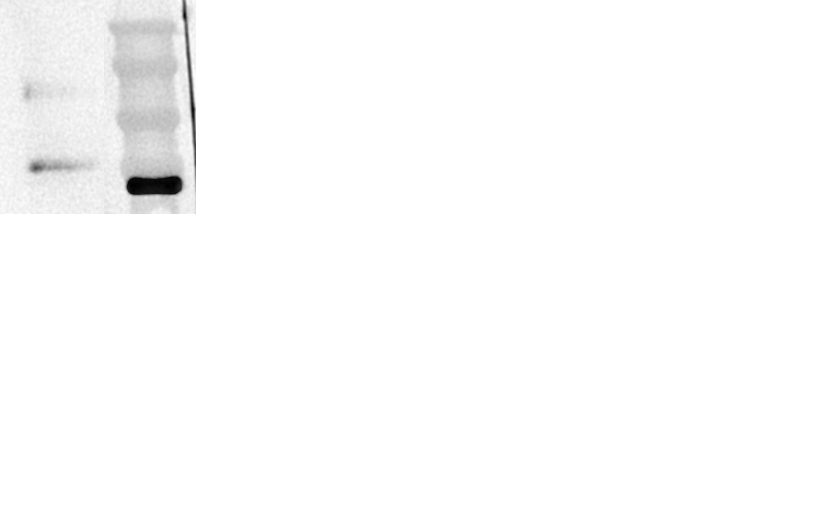

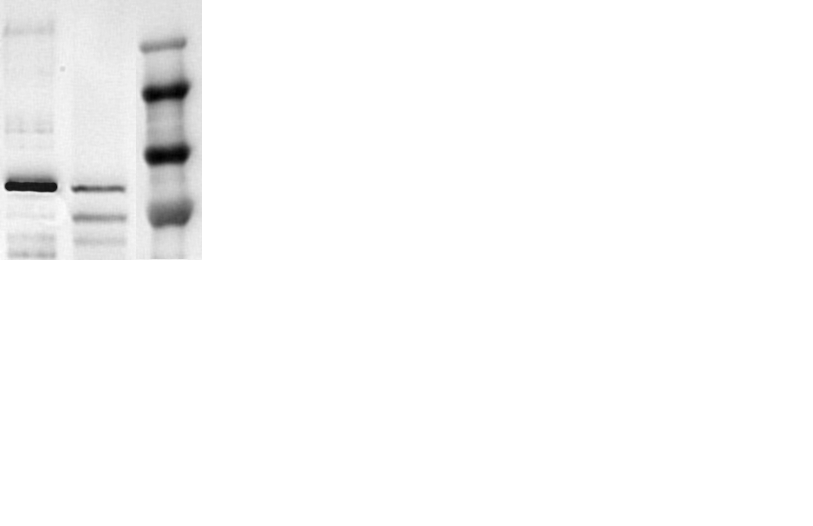


**B**

**C**

**A**

100 KDa

75 KDa

150 KDa

250 KDa
