# Supplementary material for: Salinity-Dependent Shift in the Localization of Three Peptide Transporters along the Intestine of the Mozambique Tilapia (Oreochromis mossambicus)
Source: Front Physiol. 2017 Jan 23;8:8. doi: 10.3389/fphys.2017.00008 (PMC5253378; doi:10.3389/fphys.2017.00008)

**Appendix 3**

Shared synteny of the SLC15A1 variants in tilapia and zebrafish. In both species there is a block of SLC15A1 and two flanking genes, serine/threonine-protein kinase 24 (stk24) and dedicator of cytokinesis 9 (dock9), conserved over two different chromosomes. This synteny in two distinct teleost species is an indication of shared genome duplication in their ancient common ancestor.


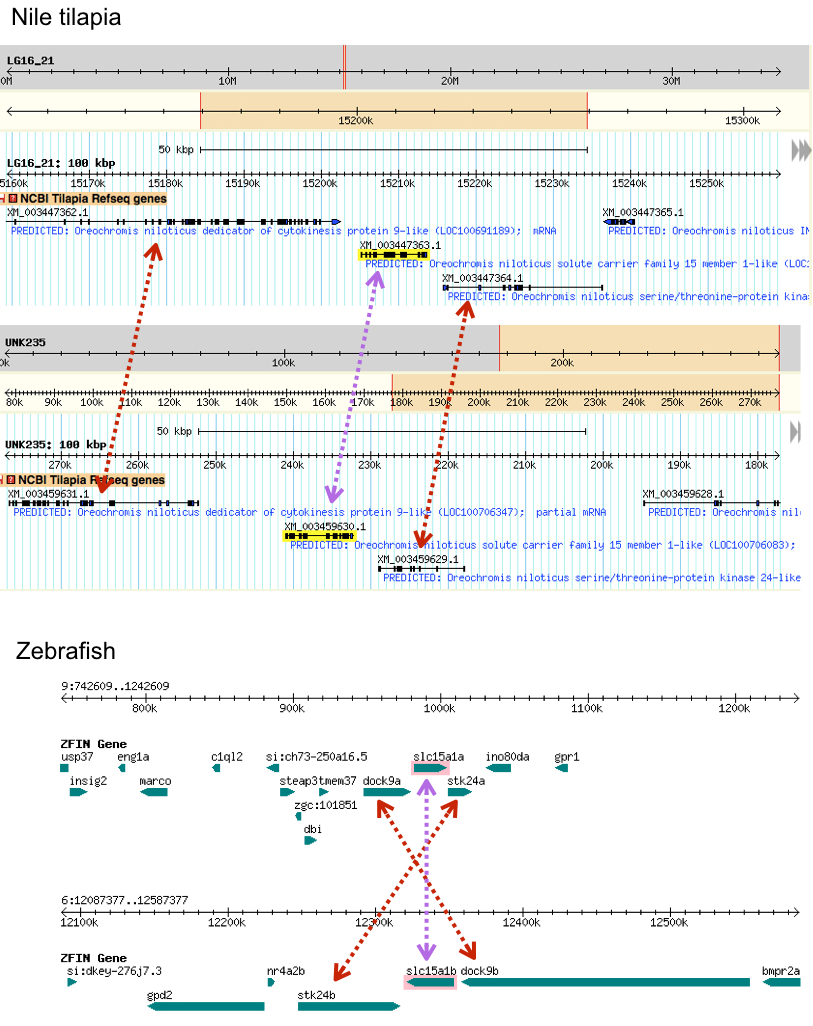

Supplement: Supplementary file 3 [file DataSheet3.DOCX]
