# Supplementary material for: Salinity-Dependent Shift in the Localization of Three Peptide Transporters along the Intestine of the Mozambique Tilapia (Oreochromis mossambicus)
Source: Front Physiol. 2017 Jan 23;8:8. doi: 10.3389/fphys.2017.00008 (PMC5253378; doi:10.3389/fphys.2017.00008)

**Appendix 4**

Relative quantification of mRNA expression of NHE3 in the anterior (A) and posterior (B) intestine and VHA in the anterior (C), middle (D) and posterior (E) intestine. Different letters indicate significant differences between time-points after feeding, except panels A and E where there was significant interaction between time and salinity. In panel B the asterisks indicate on significant difference between salinities (N=6 for each E treatment).


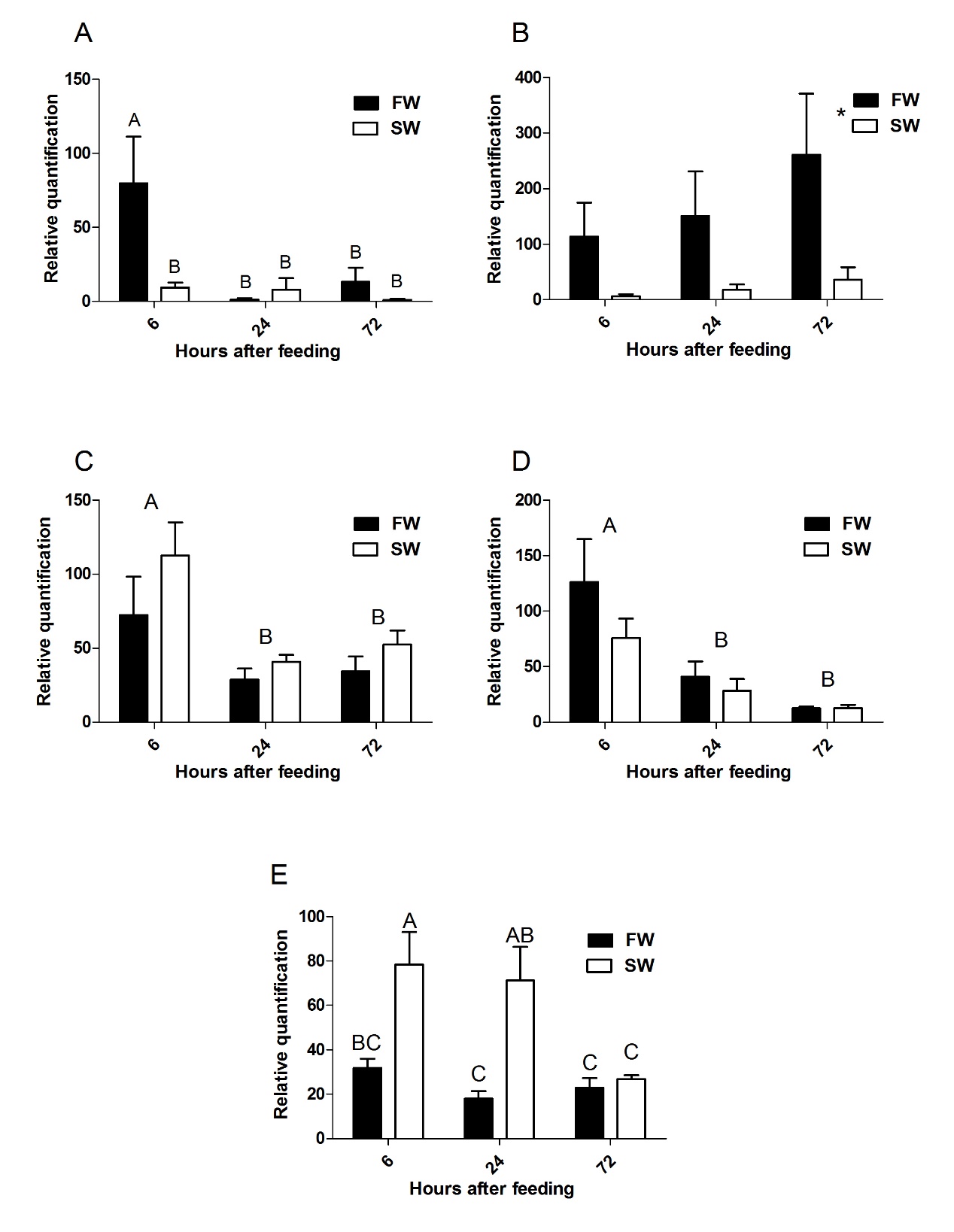

Supplement: Supplementary file 4 [file DataSheet4.DOCX]
