# Supplementary material for: Salinity-Dependent Shift in the Localization of Three Peptide Transporters along the Intestine of the Mozambique Tilapia (Oreochromis mossambicus)
Source: Front Physiol. 2017 Jan 23;8:8. doi: 10.3389/fphys.2017.00008 (PMC5253378; doi:10.3389/fphys.2017.00008)

**Appendix 5**

Immunofluorescence staining of the anterior intestine with: rabbit anti PepT1a (a), rabbit anti PepT1b (b) and rabbit anti PepT2 (c). Nuclei are stained in blue. In each: (A) negative control (no primary antibody), (B) staining with primary antibody, (C) staining with primary antibody incubated with self immunizing peptide, (D) staining with primary antibody incubated with immunizing peptide of the other variant.


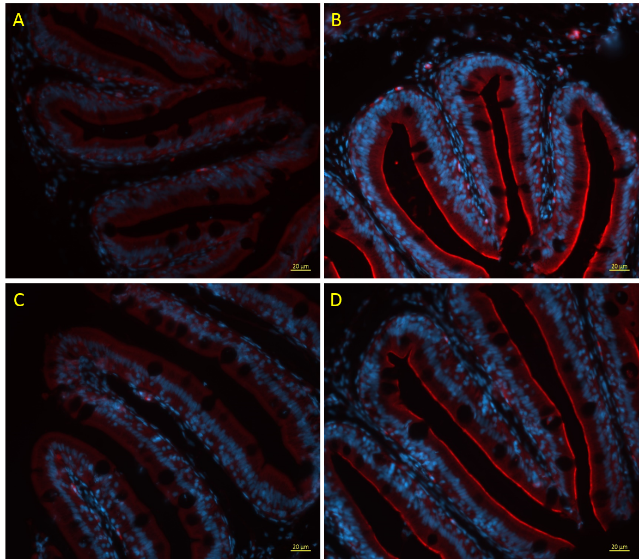

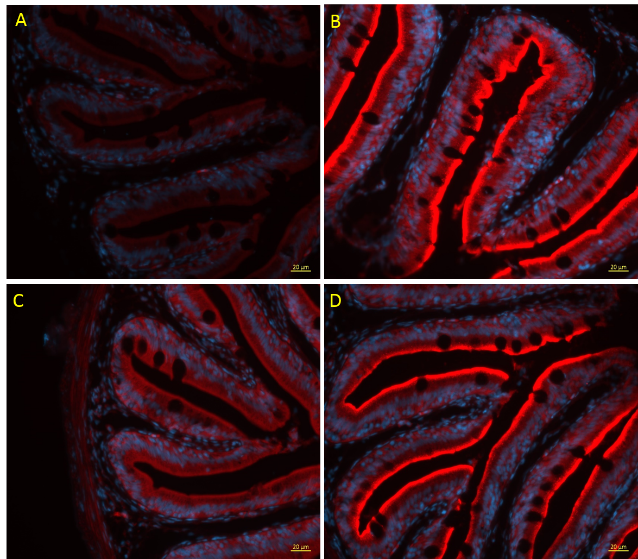


**a**

**b**

**c**


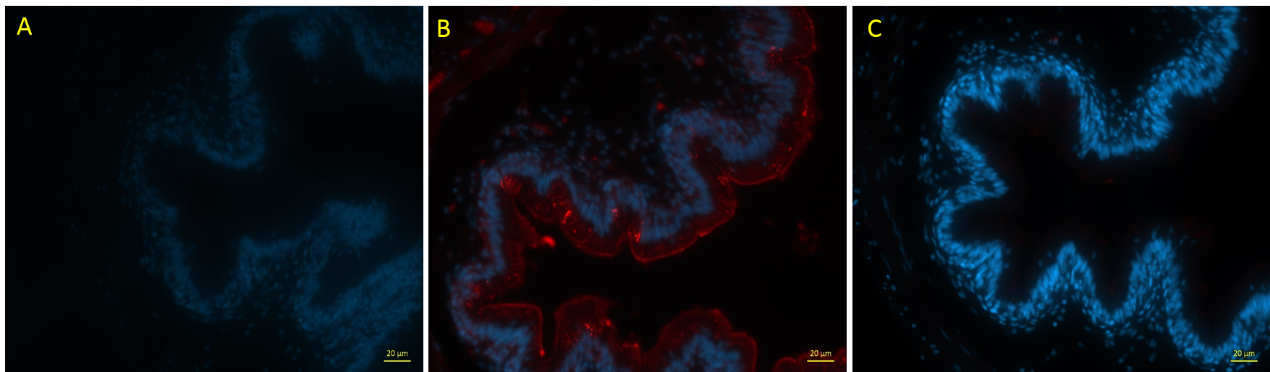

Supplement: Supplementary file 5 [file DataSheet5.DOCX]
