# Supplementary material for: Salinity-Dependent Shift in the Localization of Three Peptide Transporters along the Intestine of the Mozambique Tilapia (Oreochromis mossambicus)
Source: Front Physiol. 2017 Jan 23;8:8. doi: 10.3389/fphys.2017.00008 (PMC5253378; doi:10.3389/fphys.2017.00008)
Supplement: Supplementary file 6 [file DataSheet6.DOCX]

**Appendix 6**

Immunofluorescence staining of the anterior intestine with: rabbit anti PepT1a (A) and rabbit anti PepT1b (B), in the two salinities (Red). Nuclei are stained in blue.

**A**

**B**


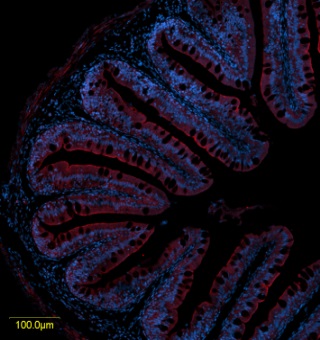

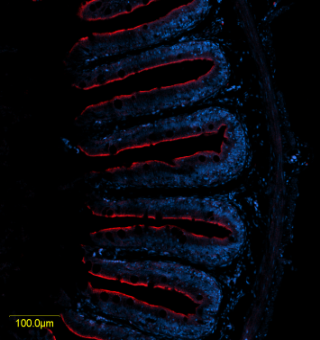

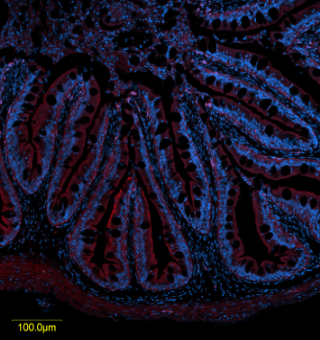

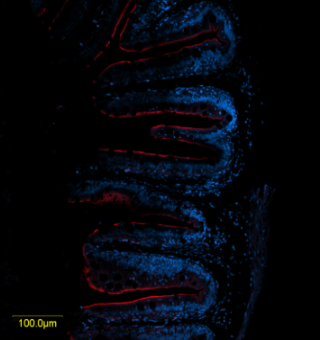


**6h**


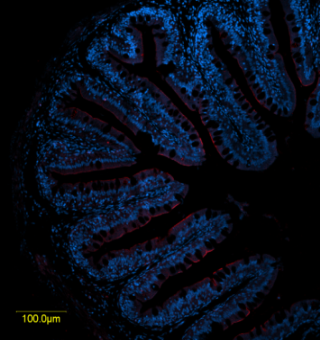


**72h**


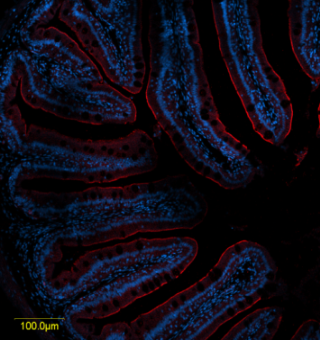


**6h**


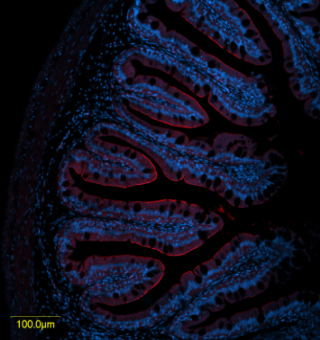


**72h**


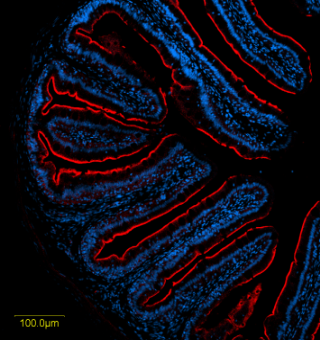


**72h**

**6h**

**6h**

**72h**

**FW**

**FW**

**FW**

**FW**

**SW**

**SW**

**SW**

**SW**
