# Supplementary material for: Salinity-Dependent Shift in the Localization of Three Peptide Transporters along the Intestine of the Mozambique Tilapia (Oreochromis mossambicus)
Source: Front Physiol. 2017 Jan 23;8:8. doi: 10.3389/fphys.2017.00008 (PMC5253378; doi:10.3389/fphys.2017.00008)
Supplement: Supplementary file 7 [file DataSheet7.DOCX]

**Appendix 7**

Trans-membrane helixes prediction for PepT sequences clustered with *O. mossambicus* PepT1a.

|  | **length** | **#TMH** | **TM1** | **TM2** | **TM3** | **TM4** | **TM5** | **TM6** | **TM7** | **TM8** | **TM9** | **TM10** | **TM11** | **TM12** | **TM13** |
| --- | --- | --- | --- | --- | --- | --- | --- | --- | --- | --- | --- | --- | --- | --- | --- |
| ***A. japonica* Pept1** | 755 | 11 | 36-53 | 63-85 | 92-114 | 134-156 | 169-191 | 206-228 | 288-305 | 334-356 | 369-388 | 622-641 | 654-676 | - |  |
| ***D. labrax* PepT** | 727 | 12 | 12-34 | 54-76 | 88-110 | 125-147 | 167-189 | 204-226 | 283-300 | 329-351 | 364-383 | 584-606 | 619641 | 646-668 |  |
| ***D. rerio* PepT1b** | 718 | 13 | 35-54 | 64-86 | 91-113 | 133-155 | 168-190 | 205-227 | 279-301 | 333-355 | 368-390 | 539-561 | 581-603 | 618-637 | 650-672 |
| ***G. aculeatus* PepT1b** | 708 | 12 | 13-35 | 55-77 | 89-111 | 121-143 | 164-186 | 206-228 | 284-301 | 331-353 | 365-384 | 584-606 | 619-641 | 651-673 |  |
| ***G. morhua* PepT** | 729 | 12 | 35-52 | 62-84 | 91-113 | 133-155 | 168-190 | 205-227 | 279-301 | 334-356 | 369-388 | 576-598 | 611-633 | 643-665 |  |
| ***O. latipes* PepT1** | 732 | 12 | 17-39 | 59-81 | 93-115 | 130-152 | 172-194 | 209-231 | 288-305 | 335-357 | 369-388 | 586-608 | 621-643 | 653-675 |  |
| ***O. niloticus* PepT1a** | 727 | 12 | 16-38 | 58-80 | 91-113 | 133-155 | 168-190 | 205-227 | 287-304 | 334-356 | 369-388 | 582-604 | 617-639 | 649-671 |  |
| ***P. flavescens* PepT1** | 729 | 13 | 16-38 | 58-80 | 92-111 | 126-148 | 169-191 | 206-228 | 287-304 | 334-356 | 369-389 | 552-573 | 586-608 | 618-637 | 650-672 |
| ***S. salar* PepT1** | 734 | 12 | 34-51 | 61-83 | 90-112 | 132-154 | 167-189 | 204-226 | 278-300 | 332-354 | 367-386 | 586-607 | 621-640 | 650-672 |  |
| ***T. nigroviridis* PepT1** | 725 | 12 | 13-35 | 60-82 | 89-111 | 131-153 | 166-188 | 203-225 | 285-302 | 332-354 | 366-385 | 585-607 | 620-639 | 649-671 |  |
| ***T. rubripes* PepT1b** | 721 | 12 | 16-38 | 58-80 | 92-114 | 129-151 | 171-193 | 208-230 | 287-304 | 333-355 | 368-390 | 587-609 | 622-644 | 654-676 |  |

Trans-membrane helixes prediction for PepT sequences clustered with *O. mossambicus* PepT1b.

|  | **length** | **#TMH** | **TM1** | **TM2** | **TM3** | **TM4** | **TM5** | **TM6** | **TM7** | **TM8** | **TM9** | **TM10** | **TM11** | **TM12** |
| --- | --- | --- | --- | --- | --- | --- | --- | --- | --- | --- | --- | --- | --- | --- |
| ***D. rerio* PepT1a** | 717 | 10 | 19-41 | 61-83 | 95-117 | 127-149 | 169-191 | 211-233 | 282-304 | 371-388 | 619-641 | 651-637 |  |  |
| ***F. heteroclitus* PepT1** | 700 | 12 | 13-32 | 52-74 | 86-108 | 123-145 | 165-187 | 202-224 | 280-298 | 327-349 | 362-381 | 582-604 | 617-636 | 646-668 |
| ***G. aculeatus* PepT1a** | 713 | 11 | 42-64 | 76-98 | 113-135 | 155-177 | 192-214 | 271-288 | 317-339 | 352-371 | 573-595 | 608-627 | 637-659 |  |
| ***O. niloticus* PepT1b** | 729 | 11 | 20-42 | 62-84 | 96-118 | 133-155 | 175-197 | 212-234 | 283-305 | 337-359 | 372-391 | 626-645 | 658-680 |  |
| ***S. nebulosus* PepT1** | 742 | 12 | 15-32 | 39-56 | 66-88 | 95-117 | 137-159 | 172-194 | 209-231 | 282-304 | 336-358 | 371-390 | 631-650 | 663-685 |
| ***T. nigroviridis* PepT1a** | 683 | 11 | 9-31 | 51-73 | 85-107 | 122-144 | 164-186 | 201-223 | 280-297 | 326-248 | 361-380 | 612-634 | 641-663 |  |
| ***T. rubripes* PepT1a** | 724 | 12 | 17-39 | 64-86 | 93-112 | 127-149 | 170-192 | 207-229 | 289-306 | 335-357 | 370-392 | 591-613 | 626-645 | 655-677 |

Trans-membrane helixes prediction for PepT sequences clustered with *O. mossambicus* PepT2.

|  | **length** | **#TMH** | **TM1** | **TM2** | **TM3** | **TM4** | **TM5** | **TM6** | **TM7** | **TM8** | **TM9** | **TM10** | **TM11** |
| --- | --- | --- | --- | --- | --- | --- | --- | --- | --- | --- | --- | --- | --- |
| ***D. rerio* PepT2** | 179 | 11 | 44-61 | 71-93 | 100-119 | 134-156 | 169-191 | 206-228 | 284-301 | 332-354 | 366-388 | 630-652 | 659-681 |
| ***O. niloticus* PepT2** | 702 | 11 | 26-43 | 53-75 | 82-104 | 109-131 | 151-173 | 188-210 | 266-283 | 313-335 | 348-370 | 610-632 | 639-661 |
