# Supplementary material for: Salinity-Dependent Shift in the Localization of Three Peptide Transporters along the Intestine of the Mozambique Tilapia (Oreochromis mossambicus)
Source: Front Physiol. 2017 Jan 23;8:8. doi: 10.3389/fphys.2017.00008 (PMC5253378; doi:10.3389/fphys.2017.00008)
Supplement: Supplementary file 8 [file DataSheet8.DOCX]

# Appendix 8

Genebank and Ensmble accession numbers for phylogenetic analysis in figure 1.

| **Name** | **Genebank** |
| --- | --- |
| *Homo sapiens* SLC15A1 | NM_005073.3 |
| *Homo sapiens* SLC15A2 | S78203.1 |
| *Macaca mulatta* SLC15A2 | NM_001032953.1 |
| *Rattus norvegicus* SLC15A1 | NM_057121.1 |
| *Rattus_norvegicu*  SLC15A2 | NM_031672.2 |
| *Mus_musculus* SLC15A2 | NM_021301.3 |
| *Sus scrofa* SLC15A1 | NM_214347.1 |
| *Gallus gallus* SLC15A1 | NM_204365.1 |
| *Gallus gallus* SLC15A2 | KF366604.1 |
| *Meleagris gallopavo* SLC15A1 | AY157977.1 |
| *Xenopus laevis slc15a2* | NM_001086929.1 |
| *Danio rerio* slc15a1b | NM_198064.1 |
| *Danio rerio* slc15a1a | XM_001919879.5 |
| *Danio rerio* slc15a2 | NM_001039828.1 |
| *Tetraodon nigroviridis* slc15a1 | JX177494 |
| *Salmo salar* slc15a1 | NM_001146682.1 |
| *Oryctolagus* *cuniculus* slc15a2 | NM_001082700.1 |
| *Oryzias* *latipes* slc15a1 | XM_004081936.1 |
| *Oryzias latipes* slc15a1 | XM_004066653.2 |
| *Carassius carassius* slc15a1 | HM453869.1 |
| *Perca flavescens slc15a1* | GQ906471.2 |
| *Dicentrarchus labrax* slc15a1 | FJ237043.2 |
| *Chionodraco hamatus* slc15a1 | AY170828.2 |
| *Gadus morhua* slc15a | AY921634.1 |
| *Sebastes nebulosus* | EU160494.1 |
| *Anguilla japonica* SLC15A1 | AB762417.1 |
| *Oreochromis niloticus* slc15a1a | XM_013267250 |
| *Oreochromis* mossambicus slc15a1a | KX034110 |
| *Oreochromis*  *niloticus* slc15a2 | XM_005475385 |
| *Oreochromis*  mossambicus slc15a2 | KX034111 |
| *Oreochromis*  niloticus slc15a1b | XM_005452882 |
| *Oreochromis* mossambicus slc15a1b | KX034112 |

| **Name** | **Ensmble** |
| --- | --- |
| *Takifugu rubripes* slc15a1b | ENSTRUT00000018670 |
| *Takifugu rubripes* slc15a1a | ENSTRUT00000044692 |
| *Tetraodon nigroviridis* slc15a1a | ENSTNIT00000017204 |
| *Gasterosteus aculeatu* slc15a1b | ENSGACT00000018320 |
| *Gasterosteus aculeatu* slc15a1a | ENSGACT00000005667 |
